# The Malarial Taint

**Published:** 1877-08

**Authors:** 


### THE MALARIAL TAINT.

A writer describing the New York Foundling Hospital, in the *Medical Journal* of that city, remarks that in malaria there is a tendency to cerebral, thoracic, and abdominal congestion, and this congestion of the viscera offers us an explanation of persistency of bronchitis and diarrhœa, complicated with cerebral symptoms. What is more important is, that it suggests a guide in therapeutics, which can be followed with marked benefit. Those who have not had their attention directed to the subject may, in cases similar to those referred to, notice that bronchitis and diarrhœa occur with and without a malarial complication. An acute malarial course will often cure an obstinate bronchitis or diarrhœa.

